# Recurrent Bacteremia with Corynebacterium Striatum After Prosthetic Valve Replacement: A Case Report

**DOI:** 10.7759/cureus.4670

**Published:** 2019-05-15

**Authors:** Mubbasher A Syed, Nikita Ashcherkin, Murtaza Sundhu, Laila Hakam, Sajjad Gul

**Affiliations:** 1 Cardiology, University of Toledo Medical Center, Toledo, USA; 2 Internal Medicine, Order of St. Francis - St. Francis Medical Center, Peoria, USA; 3 Internal Medicine, Cleveland Clinic, Fairview Hospital, Cleveland, USA

**Keywords:** infective endocarditis, recurrent bacteremia, prosthetic valve endocarditis, corynebacterium striatum

## Abstract

Infective endocarditis is typically caused by *Staphylococcus aureus (S. aureus), *coagulase-negative staphylococci and streptococci but infection with *Corynebacterium striatum (C. striatum)* is also becoming prominent. We present the case of a 65-year-old female with a recent history of the coronary artery bypass graft with bioprosthetic aortic valve replacement. The surgery was complicated by sternal wound dehiscence with methicillin-sensitive *S. aureus* (MSSA) for which she was treated for six weeks with intravenous antibiotics. Two months later, she was found to have *C. striatum* which was treated. A transesophageal echocardiogram was done as well which did not show any vegetation. She presented to the hospital with vomiting, cough, fever, and shortness of breath. She had pyuria on urinalysis and was started on empiric antibiotics after taking blood cultures. She decompensated soon after admission and was transferred to the intensive care unit where she had a pulseless ventricular tachycardia and was resuscitated but required vasopressor support. The blood cultures from admission started growing *C. striatum* again. Daptomycin was added to the empiric antibiotics and supportive care was continued, but the family decided to make her 'do not resuscitate - comfort care only'. The support was withdrawn and she passed away.

## Introduction

Infective endocarditis (IE) is an infection of the endocardial surface, which reflects the infection of the heart valves or infection of the intracardiac devices. Outcomes of infectious endocarditis are grim. Mortality rates in patients with a prosthetic or native valve IE reach as high as 20% to 25% [1]. Prosthetic valve endocarditis accounts for 10% to 30% of all causes of IE, making it a significant component of mortality in patients [2]. Although *Staphylococcus aureus (S. aureus),* coagulase-negative staphylococci and streptococci are the biggest offenders, *Corynebacterium striatum (C. striatum)* is beginning to appear more frequently as the causative agent [2]. The first published case report of prosthetic valve IE caused by *C. striatum* was published in 2002, which was found in a 72-year-old female with an aortic valve replacement [3].

## Case presentation

We present a 65-year-old female with known coronary artery disease (CAD), diabetes mellitus (DM) type II, chronic diastolic heart failure, and a recent history of coronary artery bypass grafting (CABG), along with bioprosthetic aortic valve replacement (AVR). She presented from a nursing home with a five-day history of vomiting, cough, fever, shortness of breath and reports of low pulse oximeter readings. On admission, the leukocyte count was 16,000 (range: 4,000 - 11,000 per microliter of blood), the international normalized ratio (INR) was 8.5 (range; 2.0 - 3.0 when therapeutic), and her urinalysis revealed pyuria. Blood cultures were drawn and intravenous ciprofloxacin was empirically started for the suspected urinary tract infection (UTI) and sepsis.

Chart review revealed a protracted infectious history after the CABG and AVR. The patient’s postoperative course was complicated by sternal wound dehiscence and methicillin-susceptible *S. aureus* (MSSA) bacteremia. A peripherally inserted central catheter (PICC) line was used for six weeks of intravenous vancomycin administration. Two months later, she was hospitalized with recurrent falls and her blood cultures grew *C. striatum*. The PICC line was removed and she was discharged after 10 days of intravenous vancomycin. She returned a month later with necrotizing fasciitis in the right lower limb, which culminated in an above-the-knee amputation. Once again, blood cultures showed the growth of *C. striatum*. Transesophageal echocardiography done at that time was unremarkable and did not show any vegetation. The blood cultures turned negative after another four weeks of intravenous vancomycin and the PICC line was removed.

Within hours of admission, the patient developed acute tachypnea, diaphoresis, and high-grade fever. Her antibiotic regimen was switched to intravenous ampicillin-sulbactam and she was moved to the ICU. A few hours later, she had pulseless ventricular tachycardia in the intensive care unit. After two cycles of cardiopulmonary resuscitation, including defibrillation and intubation, there was a return of spontaneous circulation. She was severely hypotensive, and hemodynamic support with vasopressors was instituted. Blood cultures drawn at admission grew *C. striatum *yet again for which daptomycin was added to her antibiotic regimen. Repeated echocardiography still did not reveal any vegetation. The patient’s family changed her code status to 'do not resuscitate - comfort care only', and soon thereafter, she was pronounced dead. 

## Discussion

Previous findings of *C. striatum *as an infectious agent were limited to native valve infective endocarditis [4]. However, a greater number of publications have since then shown that *C. striatum *is playing a bigger role than previously presumed [5-9].

Although *C. striatum *is a common flora of the skin and mucosa of the nasopharynx, it is an opportunistic pathogen in immunocompromised patients [10]. However, the question arises whether bacteremia in susceptible patients is the inciting event that causes eventual prosthetic valve IE or if prosthetic valve acts as a nidus for recurrent bacteremia. A summary of the results of the literature review regarding findings of *C. striatum *bacteremia in patients with prosthetic valves and initial imaging results is shown below in Table 1.

**Table 1:** Literature Review for Case Reports of Patients with C. striatum Prosthetic Valve Endocarditis

Reference	Age	Gender	Prosthetic Valve	Bacteremia	Initial Endocarditis Imaging Results	Clinical Outcome
de Arriba [3]	72	Female	Aortic	(+)	(+)	Death
Mashavi [7]	68	Male	Mitral	(+)	(-)	Survived
Houghton [8]	62	Female	Aortic	(+)	(-)	Survived
Stoddart [9]	72	Female	Mitral	(+)	Equivocal	Survived

As with our patient, several of the cases had positive blood cultures with a negative transthoracic echocardiogram (TTE) or transesophageal echocardiogram (TEE) findings [7-8]. The 68-year-old male described in the case reported by Mashavi et al. [7] was found to have positive blood cultures for *C. striatum *but no vegetations on TTE initially. He was then treated for pneumonia but was eventually readmitted due to the recurrence of fever. Upon the second admission, the patient grew recurrent *C. striatum *blood cultures and was found to have a 10-mm mobile mass on his prosthetic mitral valve on TTE imagining. The case report by Houghton et al. [8] of a 62-year-old woman with a history of bioprosthetic aortic valve replacement had similar clinical findings. The patient grew positive blood cultures, but TTE findings were negative for vegetations. In a case described by Stoddart et al. [9], a 72-year-old female with a history of mitral valve replacement was also found to have positive blood cultures; however, TEE findings were equivocal due to her history of culture-negative endocarditis in the past. In the first reported case of *C. striatum *infective endocarditis by de Arriba et al. [3], the patient had positive TEE findings of a 17-mm mass on her prosthetic mitral valve. However, unlike in the other cases discussed, the patient did not survive. As all of these cases highlight, positive blood cultures for *C. striatum *in patients with a history of prosthetic valve placement should be treated with urgency. Negative findings on TTE/TEE should not rule out the prosthetic valve IE. Positive cultures should be tested for antibiotic resistance. The initiation of intravenous vancomycin has shown favorable outcomes in patients. Follow-up imaging should also be performed due to the possibility of IE development after initial treatment, as was found in our case as well as others [7].

The Corynebacterium species, commonly considered contaminants in cultures, deserve attention if they grow in a patient who is immunocompromised or has a prosthesis. Our patient, with no previous history of infection with *C. striatum*, recurrently grew this organism in her blood cultures during the nine months between her valve replacement and her death. Her bacteremia resolved on two instances with intravenous antibiotics only to recur within months, suggesting a consistent source for seeding this pathogen. Despite the lack of vegetations on echocardiography, the association between recurrent bacteremia with this organism and recent prosthetic valve placement was too obvious to ignore. Due to the previous infrequency of *C. striatum *caused by IE in the literature, our suspicion of prosthetic valve IE as the source for the positive blood cultures was low. The current literature is suggestive of adding *C. striatum *to the differential in prosthetic valve IE patients. Additionally, as in the case of our patient, the diagnosis of prosthetic valve IE was elusive as the echocardiography in the first published case report was negative for vegetation [3].

## Conclusions

In a patient with a recently placed prosthetic heart valve, the growth of *C. striatum* in the blood can be indicative of endocarditis which may not manifest as vegetations on echocardiography and mandates early and aggressive intravenous antibiotics with screening for recurrence.

## References

[1] Mylonakis E, Calderwood SB (2001). Infective endocarditis in adults. N Engl J Med.

[2] Habib G, Thuny F, Avierinos JF (2008). Prosthetic valve endocarditis: current approach and therapeutic options. Prog Cardiovasc Dis.

[3] de Arriba JJ, Blanch JJ, Mateos F, Martínez-Alfaro E, Solera J (2002). Corynebacterium striatum first reported case of prosthetic valve endocarditis. J Infect.

[4] Markowitz SM, Coudron PE (1990). Native valve endocarditis caused by an organism resembling Corynebacterium striatum. J Clin Microbiol.

[5] Hascoet S, Mauri L, Claude C (2017). Infective endocarditis risk after percutaneous pulmonary valve implantation with the melody and sapien valves. JACC Cardiovasc Interv.

[6] Lee PP, Ferguson DA Jr, Sarubbi FA (2005). Corynebacterium striatum: an underappreciated community and nosocomial pathogen. J Infect.

[7] Mashavi M, Soifer E, Harpaz D, Beigel Y (2006). First report of prosthetic mitral valve endocarditis due to Corynebacterium striatum: successful medical treatment. Case report and literature review. J Infect.

[8] Houghton T, Kaye GC, Meigh RE (2002). An unusual case of infective endocarditis. Postgrad Med J.

[9] Stoddart B, Sandoe JA, Denton M (2005). Corynebacterium striatum endocarditis masquerading as connective tissue disorders. Rheumatology (Oxford).

[10] Watkins DA, Chahine A, Creger RJ, Jacobs MR, Lazarus HM (1993). Corynebacterium striatum: a diphtheroid with pathogenic potential. Clin Infect Dis.

